# Cyberchondria and Anxiety Sensitivity in Patients with Panic Disorder: A Case–Control Study

**DOI:** 10.3390/medicina62040620

**Published:** 2026-03-25

**Authors:** Kübra Orman, Tunahan Sun, Kerim Uğur, Fatma Kızılkaya

**Affiliations:** 1 Department of Psychiatry, Faculty of Medicine, Malatya Turgut Özal University, 44000 Malatya, Turkey; kubra.orman@ozal.edu.tr (K.O.); premirek@gmail.com (K.U.); 2 Department of Psychiatry, Dr. Ekrem Tok Mental Health and Diseases Hospital, 01360 Adana, Turkey; 3 Faculty of Health Sciences, Malatya Turgut Özal University, 44000 Malatya, Turkey; fatma.kizilkaya@ozal.edu.tr

**Keywords:** panic disorder, cyberchondria, anxiety sensitivity, health anxiety, reassurance-seeking behavior, online health information seeking

## Abstract

*Background and Objectives*: Cyberchondria (CYB) has been associated with health anxiety and anxiety sensitivity (AS); however, its role in panic disorder (PD) remains unclear. This study aimed to compare CYB and AS levels between patients with PD and healthy controls and to examine their associations with PD severity. *Materials and Methods*: This cross-sectional case–control study included 71 patients with PD and 69 age- and sex-matched healthy controls. Participants completed the Cyberchondria Severity Scale (CSS), Anxiety Sensitivity Index-3 (ASI-3), and Beck Anxiety Inventory (BAI). PD severity was assessed using the Panic Disorder Severity Scale (PDSS). Group comparisons were additionally conducted using analysis of covariance (ANCOVA), controlling for relevant sociodemographic and clinical variables. Pearson correlation and hierarchical multiple regression analyses were performed. *Results*: Patients with PD had significantly higher CSS (80.70 ± 22.71 vs. 60.62 ± 17.22) and ASI-3 total scores (35.66 ± 17.87 vs. 12.25 ± 10.18) than healthy controls. In the PD group, CYB was positively correlated with AS (r = 0.38, *p* < 0.01), whereas no significant association was found between CYB and PD severity (r = 0.09, *p* > 0.05). AS showed a moderate positive correlation with PD severity (r = 0.46, *p* < 0.01). In hierarchical regression analyses, CYB did not predict PD severity. Adding AS significantly increased the explained variance; however, in the final model, only general anxiety severity (BAI) remained a significant predictor of PD severity. *Conclusions*: Patients with PD exhibit elevated levels of CYB and AS, which are positively associated with each other. Nevertheless, PD severity appears to be primarily driven by general anxiety symptoms rather than CYB. These findings suggest that CYB may represent a parallel maladaptive coping behavior rather than a direct determinant of symptom severity, with potential implications for assessment and intervention. Longitudinal studies are warranted to clarify causal relationships.

## 1. Introduction

Panic Disorder (PD) is a type of anxiety disorder characterized by recurring unexpected panic attacks and a persistent state of anxiety regarding the possible consequences of these attacks. Panic attacks are usually accompanied by at least four of the following somatic and cognitive symptoms: palpitations, sweating, trembling, shortness of breath, feeling of suffocation, chest pain, dizziness, lightheadedness, fear of losing control, fear of death, and derealization [1]. Although it may vary by geographic location, the lifetime prevalence of PD is estimated at 1.7% [2]. PD is associated with substantial impairments in functioning and quality of life [3].

Anxiety sensitivity (AS), on the other hand, is defined as an excessive fear that anxiety-related sensations and symptoms may lead to harmful physical, psychological, or social consequences [4]. Studies have also reported that high AS scores predict the risk of panic attacks and susceptibility to PD [5], may be associated with the onset and persistence of PD symptoms [6], and may be related to the severity of PD [7]. Additionally, it has been found that patients with PD have higher AS levels compared to healthy individuals [8].

With the widespread use of the Internet, new digital health-related phenomena have been identified in the literature. Cyberchondria (CYB) is a digital health issue characterized by excessive and repetitive online searches for health information to manage rising health concerns or anxiety [9]. A study found that 64.3% of participants reported searching for health information online when they experienced acute symptoms [10]. CYB is particularly relevant in countries with widespread internet access. In Türkiye, internet use has increased substantially over the past decade. According to the Turkish Statistical Institute, internet use among individuals aged 16–74 years increased from 88.8% in 2024 to 90.9% in 2025 [11]. In addition, the proportion of households with internet access at home reached 95.5% in 2023, up 1.4 percentage points from the previous year [12]. In such a highly connected digital environment, searching for health-related information online has become a common behavior among the general population.

However, easy access to online health information has also been associated with increased health anxiety and CYB in some individuals [13,14]. Although the internet provides great convenience in terms of accessing medical information, for individuals with pre-existing anxiety or heightened health concerns, online searches may paradoxically exacerbate rather than alleviate their worries [15].

### Theoretical Background

AS has been widely recognized as a central vulnerability factor in the development and maintenance of PD [5,6]. Cognitive models of PD propose that individuals with elevated AS tend to catastrophically misinterpret benign bodily sensations (such as palpitations or dizziness) as signs of imminent physical or psychological danger, which may trigger panic attacks and perpetuate panic-related fear [16]. In this context, heightened sensitivity to bodily sensations may lead individuals to engage in reassurance-seeking behaviors, such as searching for health-related information online, in an attempt to reduce perceived health threats.

In parallel, CYB has been conceptualized as a maladaptive pattern of online health information seeking characterized by repeated searches for medical information driven by health anxiety, intolerance of uncertainty, and reassurance-seeking motives [17,18,19]. Although individuals often seek online health information to reduce uncertainty about bodily symptoms, repeated exposure to ambiguous or alarming medical information may paradoxically increase anxiety and reinforce catastrophic interpretations of bodily sensations [15,20].

Given that patients with PD frequently experience distressing bodily sensations and elevated AS [8], they may be particularly prone to engaging in repetitive online health information-seeking behaviors in an attempt to obtain reassurance or explanations for their symptoms. Therefore, examining the relationship between CYB and AS in patients with PD may help clarify the psychological mechanisms underlying excessive online health information-seeking behaviors in this population.

In the literature, CYB is generally associated with health anxiety, hypochondriasis, and obsessive–compulsive disorder [21,22]. However, to our knowledge, no study has specifically investigated the relationship between CYB and PD. Therefore, the present study aimed to examine the relationship between CYB and AS in patients with PD and to compare CYB and AS levels between patients with PD and healthy controls to address an existing gap in the literature. When patients with PD experience physical symptoms associated with panic (such as palpitations, dizziness, and shortness of breath), they may turn to the Internet seeking reassurance or explanations and encounter alarming information that reinforces catastrophic interpretations and perpetuates the panic cycle.

The following hypotheses guided this study:

**H1.**  *Patients with PD would exhibit higher levels of CYB and AS than healthy controls.*

**H2.**  *CYB would be positively correlated with AS in patients with PD.*

**H3.**  *Higher CYB levels would be associated with greater PD severity in patients with PD.*

## 2. Materials and Methods

### 2.1. Participants

This study was designed as a cross-sectional case–control study. The study sample consisted of patients who visited the Psychiatry Outpatient Clinic at Malatya Training and Research Hospital and were diagnosed with PD according to the Diagnostic and Statistical Manual of Mental Disorders, Fifth Edition (DSM-5) criteria by the same psychiatrist (first author), as well as a healthy control group with no psychiatric diagnosis. The patient group was included in the study using sequential sampling from cases presenting to the psychiatric outpatient clinic. The control group was formed by individuals selected voluntarily from among hospital staff and patients’ relatives, who were similar to the patient group in terms of age and sex.

The inclusion criteria for the study were age between 18 and 65 years and cognitive ability to understand and respond to the scales. To be included in the patient group, participants had to meet DSM-5 diagnostic criteria for PD. To be included in the control group, participants must have no current or past history of psychiatric diagnosis.

The exclusion criteria for both groups were defined as any psychiatric diagnosis other than PD (major depressive disorder, generalized anxiety disorder, anxiety disorders other than agoraphobia, psychotic disorders, or bipolar disorders), a history of neurodevelopmental disorders, neurological disease or significant cognitive impairment that could interfere with the administration of the scale, intellectual disability, delirium, dementia, and active alcohol or substance use disorder. Participants who did not complete the evaluation process or who filled out the scale forms incompletely or incorrectly were excluded from the analysis. PD is frequently seen alongside depression and other anxiety disorders; however, these comorbidities can have strong and independent effects, particularly on anxiety severity, AS, and health-related concerns. Therefore, to obtain more methodologically robust and interpretable results, all comorbid psychiatric diagnoses identified using the Structured Clinical Interview for DSM-5—Clinician Version (SCID-5-CV) were excluded, and the assessment of PD-specific psychometric properties was targeted. Agoraphobia was not included among the exclusion criteria because of its close clinical and nosological relationship with PD; it was additionally recorded as a comorbid diagnosis in the patient group.

Ninety patients with PD were included at the beginning of the study. Thirteen patients were excluded due to additional psychiatric diagnoses other than PD, two due to active substance use disorder, three due to incomplete scale completion, and one due to incorrect scale completion. After applying the exclusion criteria, 71 patients diagnosed with PD were included in the study.

**Figure 1:**
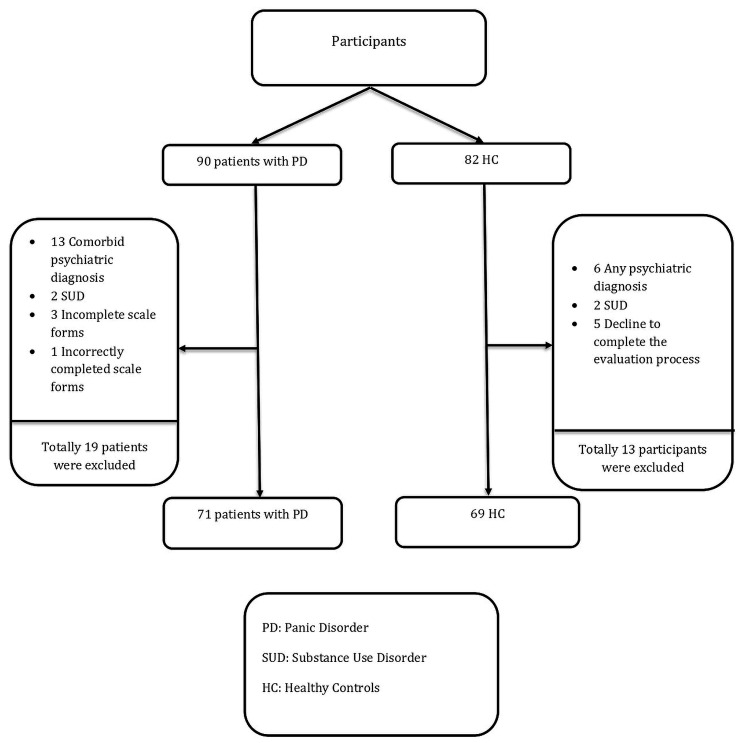
Flowchart of participant selection, exclusion criteria, and final sample inclusion for patients with panic disorder and healthy control groups.

In the control group, 82 participants were initially enrolled. Six participants were excluded from the study due to psychiatric diagnoses, two participants due to active substance use disorder, and five participants due to their unwillingness to complete the assessment process. After applying these exclusion criteria, 69 healthy controls were included in the study. The participant selection process, exclusion criteria, and final sample inclusion are illustrated in Figure 1.

### 2.2. Power Analysis

Power analysis was performed using G*Power software (version 3.1.9.2; Heinrich Heine University Düsseldorf, Düsseldorf, Germany) to determine the required sample size. For comparisons between two independent groups, a medium effect size (Cohen’s d = 0.50), a significance level of 0.05, and 80% test power (1 − β = 0.80) were used as the basis for sample size calculation. Based on these parameters, the minimum sample size per group was 64. The study included 71 participants in the patient group and 69 in the control group. Both groups met the minimum sample size requirement, and the study had sufficient statistical power.

### 2.3. Procedure

All participants were provided with detailed information about the purpose, scope, and procedures of the study, and written informed consent was obtained. Data were collected at the psychiatry outpatient clinic using a structured, face-to-face assessment. All evaluations were conducted in a quiet consultation room of the psychiatry outpatient clinic under standardized conditions and during regular outpatient working hours.

Individual clinical interviews, averaging 30–45 min, were conducted by the first author (the same psychiatrist) with participants in the patient group. The assessment procedure followed a standardized sequence. First, a clinical diagnostic evaluation was conducted in accordance with DSM-5 criteria. Subsequently, comorbid psychiatric diagnoses were systematically assessed using SCID-5-CV.

Participants diagnosed with any psychiatric disorder other than PD were excluded from the study. Following the clinical interview, sociodemographic and clinical data forms were completed, and the psychometric assessment was initiated. After completion of the diagnostic assessment, the clinician evaluated PD severity using the Panic Disorder Severity Scale (PDSS). Within the scope of psychometric evaluation, participants then completed the self-report questionnaires, including the Cyberchondria Severity Scale (CSS), Beck Anxiety Inventory (BAI), and Anxiety Sensitivity Index-3 (ASI-3). No intervention was provided to guide participants’ responses during administration of the self-report scales, and participants completed the questionnaires individually in the same clinical setting.

The control group was also confirmed to have no history of psychiatric diagnosis using the SCID-5-CV, after which the sociodemographic data form and psychometric scales were administered using the same standard procedure. Thus, the assessment sequence for both groups consisted of diagnostic evaluation, clinician-rated PD severity assessment, and subsequent administration of the self-report scales. All evaluations were conducted in a quiet and appropriate clinical setting. Incomplete or incorrectly filled-out scales were excluded from the assessment.

### 2.4. Data Collection

#### 2.4.1. Sociodemographic Data Form

Participants’ sociodemographic characteristics, including age, gender, marital status, educational level, income level, family structure, region of residence, family history of psychiatric illness, and smoking habits, were assessed using a structured sociodemographic data form.

#### 2.4.2. Clinical Data Form

Clinical data were collected using a structured data form. The age at disease onset, disease duration, time between diagnosis and initiation of treatment, presence of agoraphobia as a comorbid diagnosis, and use of psychotropic medications during the assessment were recorded in the patient group. Panic attack symptoms were assessed using a structured checklist based on the panic attack symptoms listed in the DSM-5 diagnostic criteria and were recorded as present or absent.

#### 2.4.3. Panic Disorder Severity Scale (PDSS)

It is a measure used to assess PD severity and is administered by clinicians. The past month was used as the reference period. The scale consists of seven subscales: frequency of panic attacks, distress during attacks, severity of anticipatory anxiety, agoraphobic fear/avoidance, fear/avoidance of situations associated with panic attacks, impairment/disruption in work functioning due to PD, and impairment/disruption in social functioning due to PD. Each item is scored on a scale of 0–4, and the total score is then calculated. Higher scores indicate more severe PD. Shear et al. conducted a validity and reliability study of the original version of the scale [23]. Monkul et al. conducted a validity and reliability study of the scale in Turkish, demonstrating that it is a reliable and valid tool for assessing PD severity [24].

#### 2.4.4. Cyberchondria Severity Scale (CSS)

This is a 33-item self-report scale developed to assess CYB levels. It consists of five subscales: Compulsion, Distress, Excessiveness, Reassurance-seeking, and Mistrust of the doctor. Items are scored on a scale of 0–4, and the subscale scores can be evaluated separately. Higher scores indicate a higher level of CYB in the sample. The original form of the scale was developed by McElroy and Shevlin [25]. The Turkish validity and reliability study by Selvi et al. reported that the scale has high internal consistency and is a valid and reliable tool for assessing CYB [26].

#### 2.4.5. Beck Anxiety Inventory (BAI)

It is a 21-item self-report scale that assesses the severity of anxiety symptoms in the past week. Items are scored from 0 to 3, and the total score is calculated. The total score ranges from 0 to 63, with higher scores indicating greater anxiety. The original inventory was developed by Beck et al. [27]. Ulusoy et al. conducted a Turkish validity and reliability study and demonstrated that the Turkish version of the scale is reliable and valid for assessing anxiety symptoms [28].

#### 2.4.6. Anxiety Sensitivity Index-3 (ASI-3)

The Anxiety Sensitivity Index-3 is a self-report scale that assesses AS. The scale comprises 18 items across three subscales: physical, cognitive, and social. Each item is scored on a scale of 0–4, and the total score is then calculated. A high total score indicates high AS. Taylor et al. conducted a validity and reliability study of the original version of the scale [29]. A validity and reliability study was conducted in Turkish, and it was reported that the Turkish version of the scale demonstrated psychometric properties across total scores and subdimensions [30].

### 2.5. Ethics

The study was approved by the Malatya Turgut Özal University Health Sciences Scientific Research Ethics Committee (protocol number: 2024/400). This study was conducted in accordance with the Declaration of Helsinki. Participation in the study was voluntary, and participants received no financial compensation or incentives.

### 2.6. Statistical Analysis

IBM SPSS Statistics (version 23.0; IBM Corp., Armonk, NY, USA) and Stata/IC (version 14.2; StataCorp LLC, College Station, TX, USA) were used for statistical analysis. The distribution characteristics of continuous variables were assessed using skewness and kurtosis values, and it was assumed that the normal distribution assumption was satisfied if these values fell within ±2. Descriptive statistics are presented as mean ± standard deviation and median (minimum–maximum) for continuous variables and number and percentage for categorical variables. Group differences were examined using analysis of covariance (ANCOVA) to compare patients and healthy controls while controlling for potential confounding variables, including educational level, employment status, income level, smoking status, and family history of mental illness. In the analysis of categorical variables, Pearson’s chi-square test, Fisher’s exact test, and Monte Carlo simulation were used when appropriate. Effect sizes for ANCOVA were reported using partial eta squared (η^2^), with values of 0.01, 0.06, and 0.14 indicating small, medium, and large effect sizes, respectively.

The direction and strength of the linear relationships between the scales in the patient group were assessed using Pearson’s correlation analysis. Hierarchical multiple linear regression analysis was applied to the patient group to determine the variables predicting the PDSS-Total score. The basic assumptions were checked before the regression analysis. Additionally, independent samples *t*-tests were conducted within the patient group to compare CYB levels according to agoraphobia status. In all statistical analyses, the significance level was set at *p* < 0.05.

### 2.7. Artificial Intelligence Statement

The authors declare that generative artificial intelligence tools developed by OpenAI (ChatGPT, version GPT-5.3) were used solely for language editing and grammatical improvement of the manuscript. These tools did not contribute to the study design, data analysis, interpretation of results, or generation of scientific content. All authors reviewed and approved the final version of the manuscript and take full responsibility for the content.

## 3. Results

Statistically significant differences were found between the patient and control groups in terms of education level, employment status, income level, smoking habits, and family history of mental illness (*p* < 0.05). The sociodemographic characteristics of the patient and control groups are presented in Table 1.

The mean age at disease onset in the patient group was 25.51 ± 9.10 years. The mean disease duration was 23.03 ± 71.83 months, and the mean time from diagnosis to treatment initiation was 5.72 ± 6.83 months. In the patient group, agoraphobia was present as a comorbid diagnosis in 23 patients (32.4%) and absent in 48 patients (67.6%). Additionally, 19 patients (26.8%) were receiving psychotropic medication at the time of assessment, whereas 52 patients (73.2%) were not receiving any psychotropic medication. The distribution of panic attack symptoms in the patient group is presented in Table 2.

Adjusted comparisons between patients with PD and healthy controls are presented in Table 3. After controlling for potential confounding variables, including education level, employment status, income level, smoking status, and family history of mental illness, patients with PD had significantly higher scores on ASI-3 Total, CSS-Total, and BAI-Total compared with healthy controls (ASI-3 Total: F(1, 133) = 41.57, *p* < 0.001, partial η^2^ = 0.238; CSS-Total: F(1, 133) = 13.01, *p* < 0.001, partial η^2^ = 0.089; BAI-Total: F(1, 133) = 131.82, *p* < 0.001, partial η^2^ = 0.520).

Importantly, these group differences remained statistically significant after adjustment for all covariates. None of the demographic covariates showed a significant effect on ASI-3 Total or BAI-Total scores, whereas smoking status was the only covariate significantly associated with CSS-Total scores (*p* = 0.033). Regarding subscales, employment status (*p* = 0.020) and smoking status (*p* = 0.041) had significant effects on ASI-3 Cognitive scores. Smoking status significantly influenced CSS-Compulsion (*p* = 0.002), while family history of mental illness significantly affected CSS-Mistrust (*p* = 0.007). Education level, although significantly different between groups, did not have a significant effect on the primary outcome measures in the adjusted models.

The Pearson correlations between the scales are presented in Table 4. There were significant positive correlations between the ASI-3 subscales and the ASI-3 Total score (r = 0.56–0.93, *p* < 0.01). ASI-3 Total was positively correlated with CSS-Total (r = 0.380, *p* < 0.01) and several CSS subscales, including CSS-Compulsion (r = 0.342, *p* < 0.01), CSS-Distress (r = 0.410, *p* < 0.01), and CSS-Excessiveness (r = 0.283, *p* < 0.05). However, no significant correlations were found between ASI-3 Total and CSS-Reassurance or CSS-Mistrust.

PDSS-Total was positively correlated with ASI-3 Total (r = 0.463, moderate) and BAI-Total (r = 0.571, strong). In contrast, the relationship between CSS-Total and PDSS-Total was weak and not statistically significant (r = 0.09, *p* > 0.05).

**Figure 2:**
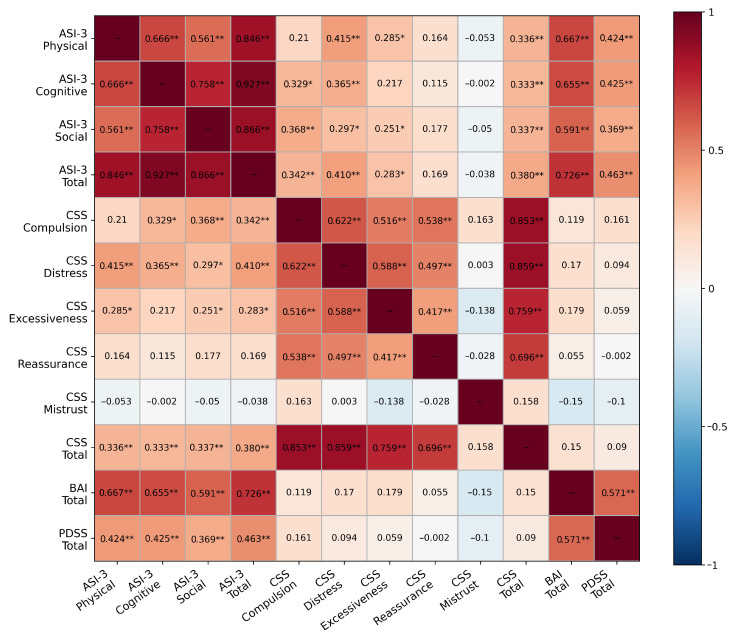
Heatmap illustrating the correlations between anxiety sensitivity dimensions, cyberchondria subscales, and anxiety and panic disorder severity scale scores in the patient group. Warmer (red) colors represent positive correlations and cooler (blue) colors represent negative correlations. Increasing color intensity corresponds to stronger correlation coefficients. ASI-3 = Anxiety Sensitivity Index-3; CSS = Cyberchondria Severity Scale; BAI = Beck Anxiety Inventory; PDSS = Panic Disorder Severity Scale. ASI-3 Physical, ASI-3 Cognitive, and ASI-3 Social represent the physical, cognitive, and social subscales of the Anxiety Sensitivity Index-3, respectively. CSS-Compulsion, CSS-Distress, CSS-Excessiveness, CSS-Reassurance, and CSS-Mistrust represent the corresponding subscales of the Cyberchondria Severity Scale. * *p* < 0.05; ** *p* < 0.01.

To enhance the readability and visual interpretation of the correlation patterns, the associations between AS, CYB, anxiety, and PDSS scores are also presented as a heatmap in Figure 2, with numerical correlation coefficients reported in Table 4.

Hierarchical regression analysis was conducted to examine predictors of PD severity. The results are presented in Table 5.

In Model 1, demographic variables (age, employment status, smoking status, and family history of mental illness) did not significantly predict PD severity (R^2^ = 0.098, *p* = 0.139).

In Model 2, the addition of CYB severity (CSS-Total) did not significantly improve the model (β = 0.063, *p* = 0.606; ΔR^2^ = 0.004).

In Model 3, the inclusion of AS (ASI-3 Total) significantly increased the explained variance (R^2^ = 0.279, ΔR^2^ = 0.177, *p* = 0.001), and ASI-3 Total emerged as a significant predictor (β = 0.477, *p* < 0.001).

In Model 4, anxiety severity (BAI-Total) was added and emerged as the strongest predictor of PD severity (β = 0.486, *p* = 0.002), further increasing the explained variance (R^2^ = 0.379, ΔR^2^ = 0.100, *p* < 0.001). In the final model, ASI-3 Total and CSS-Total were no longer significant predictors. All regression models were adjusted for demographic variables, and no multicollinearity issues were detected (VIF < 5).

To further explore the clinical characteristics of PD, CYB levels were compared between patients with and without agoraphobia using independent samples *t*-tests. The results are presented in Table 6. No statistically significant differences were observed between patients with and without agoraphobia in CSS-Total or any CSS subscale scores (all *p* > 0.05).

## 4. Discussion

To the best of our knowledge, this is the first study to examine the relationship between CYB and PD. The most important finding of this study was that patients with PD had significantly higher CYB levels than healthy controls. It has been reported that patients with PD often interpret the physical symptoms they experience during panic attacks as signs of a serious illness and frequently seek medical reassurance [31]. Studies have reported that patients with PD have significantly higher levels of health anxiety than healthy controls [32,33]. Given that CYB is defined as excessive and repetitive searching for health information online in relation to increasing health concerns [9], this may explain the high CYB levels among patients with PD in the present study.

Moreover, patients with PD exhibit emotional, cognitive, and behavioral response patterns that overlap with those observed in hypochondriasis [31]. In this context, considering that CYB is most frequently addressed in the context of hypochondriasis and health anxiety [22], the excessive focus on bodily sensations seen in PD, the interpretation of these sensations as signs of a serious illness, and the accompanying reassurance-seeking behaviors manifesting through online health information searches may contribute to increased levels of CYB.

In the present study, patients with PD had significantly higher AS levels than those in the control group. Additionally, a positive correlation was found between AS and PD severity in patients with PD. Similarly, Yang et al. reported that patients with PD had higher AS levels than healthy individuals [8]. McNally showed that high AS levels are a risk factor for panic attacks and PD [34]. Sandin et al. reported that the physical and cognitive subscales of the AS are associated with PD severity [7]. From a theoretical perspective, these findings are consistent with Clark’s cognitive model of panic, which posits that panic attacks arise from the catastrophic misinterpretation of bodily sensations [16,35]. In this framework, AS contributes to heightened vigilance toward bodily sensations, increasing the likelihood that benign physiological changes are perceived as threatening. As a result, individuals with high AS are more likely to interpret such sensations as dangerous, thereby triggering panic attacks and maintaining the panic cycle [7,36].

The present study also found a positive correlation between CYB and AS in patients with PD. Studies conducted in non-clinical samples have also reported a significant association between AS and CYB and suggested that AS may be a risk factor for the development of CYB [37]. Consistent with these findings, Abu Khait et al. also reported a positive relationship between CYB and AS [38]. A meta-analysis has further demonstrated that CYB is a distinct construct associated with AS [20]. This relationship may be explained by the tendency of individuals with high AS to perceive bodily sensations as potentially dangerous, which in turn reinforces reassurance-seeking behaviors aimed at reducing health-related uncertainty. Online health information search behaviors may contribute to the maintenance of CYB as an easily accessible form of this reassurance-seeking process [17,34,37].

On the other hand, CYB did not significantly predict PD severity in the hierarchical regression analysis. Consistently, no significant association was found between CYB and PD severity in the correlation analyses. CYB has been theoretically associated with repetitive online health searches and reassurance-seeking behaviors that predominantly arise in the context of health anxiety or hypochondriasis [17]. In this context, CYB may reflect maladaptive cognitive–behavioral processes related to health anxiety and reassurance seeking, rather than the severity of panic symptoms per se. Therefore, to better explain the role of CYB in PD, further studies are needed that include variables such as health anxiety, functional impairment, avoidance behaviors, and the long-term clinical course beyond attack severity.

From a cognitive–behavioral perspective, this pattern may be understandable, as PD severity is more closely linked to the catastrophic misinterpretation of bodily sensations and fear of anxiety-related symptoms than to online health-searching behavior per se. Contemporary models of PD emphasize that AS amplifies the perceived dangerousness of bodily sensations, thereby intensifying panic-related fear and symptom severity [16,39]. In contrast, CYB is more accurately conceptualized as a maladaptive reassurance-seeking behavior driven by health anxiety and intolerance of uncertainty, often supported by metacognitive beliefs that promote repetitive online symptom checking in an attempt to reduce perceived health threats [9,40]. Accordingly, CYB may accompany PD as a parallel maladaptive coping behavior without necessarily contributing directly to the severity of panic symptoms.

In addition, the cultural and contextual characteristics of the study population may contribute to the interpretation of the observed findings. In Türkiye, several studies have demonstrated that health-related internet use and CYB-related behaviors are influenced by factors such as health anxiety, e-health literacy, and patterns of online information seeking, within a rapidly expanding digital environment characterized by increasing internet access and use of online health information [11,12]. For instance, research conducted among women in Türkiye has shown that CYB levels are positively associated with health anxiety and e-health literacy, suggesting that individuals who frequently seek health information online may be more vulnerable to anxiety-provoking interpretations of medical information [41]. Furthermore, studies conducted in Turkish populations have highlighted that limited health literacy remains a significant public health issue, potentially increasing susceptibility to misinterpretation of online health information and heightened health-related anxiety [42]. In this context, the CYB behaviors observed in the present study may reflect broader sociocultural patterns of digital health information seeking, rather than being directly linked to the clinical severity of PD symptoms.

In hierarchical regression analysis, a significant increase in explained variance was observed when AS was added to the model alongside CYB. AS is a cognitive construct that reflects a tendency to interpret anxiety-related sensations and physical symptoms as harmful [4]. In this context, the findings suggest that cognitive vulnerability factors, such as AS, play a more prominent role than behavioral patterns, such as online health information seeking, in explaining PD severity. These results indicate that psychological mechanisms contribute to the explanation of PD severity beyond demographic and health-related background variables.

However, with the addition of BAI, which reflects the severity of general anxiety symptoms, the effect of AS was no longer statistically significant, and overall symptom severity emerged as the primary predictor of PD severity. In the present study, hierarchical regression analysis identified BAI as a significant predictor of PD severity in the final model, whereas AS and CYB were not. This finding may be explained by the content overlap between BAI and panic-related somatic symptoms. A substantial proportion of BAI items (e.g., palpitations, shortness of breath, feeling of suffocation, trembling) overlap with core symptoms of panic attacks, and previous studies have shown that the BAI may capture panic-related symptomatology rather than general anxiety alone [43]. Therefore, when BAI is included in the regression model, it may account for a large proportion of variance in PD severity, reducing the apparent contribution of AS. In this context, BAI may function as a proximal indicator of current symptom burden, whereas AS represents a more distal cognitive vulnerability factor.

In an additional analysis, patients with and without agoraphobia were compared in terms of CYB severity, and no significant differences were observed across CSS total and subscale scores. These findings suggest that CYB-related behaviors may not be directly associated with the presence of agoraphobia, but rather reflect broader cognitive and anxiety-related processes, such as AS and health anxiety. However, given the cross-sectional design and limited sample size, the potential impact of agoraphobia on the course and clinical significance of CYB cannot be fully determined. Future longitudinal studies are needed to clarify these relationships.

### 4.1. Limitations

The present study has several limitations. First, because the study is cross-sectional, causal inferences cannot be drawn about the relationships among CYB, AS, and PD severity.

Secondly, recruiting patients with PD from a single center may limit the generalizability of the findings. Given that internet usage may be influenced by cultural and socioeconomic factors, the findings should be validated in diverse samples and multicenter studies. In addition, the concept of CYB inherently assumes access to digital technologies and the internet. Therefore, in populations with limited internet access due to socioeconomic, geographic, age-related, or cultural factors, reassurance-seeking behaviors related to health anxiety may manifest through alternative behavioral patterns rather than online health information searches. For example, individuals may engage in repeated medical consultations (“doctor shopping”), excessive reliance on printed medical materials, persistent reassurance-seeking from family members or friends, or consultation with traditional or alternative healers in an attempt to reduce perceived health threats. These culturally and contextually shaped reassurance-seeking behaviors may represent functional equivalents of CYB in technologically underserved populations. Importantly, similar to CYB-related online reassurance seeking, such offline behaviors may also be reinforced by elevated AS, as individuals who are highly sensitive to bodily sensations may repeatedly seek confirmation about the meaning and potential consequences of their symptoms. From a cognitive behavioral perspective, these reassurance-seeking behaviors, whether online or offline, may contribute to the maintenance of panic-related fear by preventing individuals from disconfirming catastrophic interpretations of bodily sensations. Consequently, the relationship between AS and PD severity may manifest through different reassurance-seeking pathways depending on the cultural and technological context in which individuals attempt to manage health-related uncertainty. Future cross-cultural studies, including populations with varying levels of digital access, may help clarify how such culturally shaped reassurance-seeking behaviors interact with AS and PD severity.

Thirdly, CYB, AS, and overall anxiety levels were evaluated using self-report scales (CSS, ASI-3, and BAI). While these scales are valid and reliable tools, it should be noted that self-report measures may be susceptible to response bias.

Fourth, comorbid psychiatric diagnoses such as major depressive disorder and other anxiety disorders, which often accompany PD, were excluded for methodological reasons. Although this approach enabled a more precise assessment of PD-specific psychometric characteristics, it may limit the generalizability of the findings to real-world clinical populations, where comorbidity is common.

Finally, statistically significant differences were found between the patient and control groups for certain sociodemographic variables, including educational level, employment status, and income level. Although these variables were statistically controlled in the ANCOVA analyses, residual confounding cannot be entirely ruled out. Future studies with better-matched samples or larger datasets are needed to further validate these findings.

Additionally, due to the limited sample size, it was not feasible to include all potential covariates simultaneously in the hierarchical regression models. In particular, education level, although included as a covariate in the ANCOVA analyses, could not be incorporated into the regression models. Therefore, the regression analyses were restricted to the theoretically most relevant variables.

### 4.2. Clinical Implications and Future Research Directions

From a clinical perspective, elevated CYB levels may also have important implications for the therapeutic process in patients with PD. Repetitive online health information seeking may function as a form of reassurance-seeking behavior that temporarily reduces anxiety but ultimately maintains health-related threat beliefs, symptom monitoring, and distress. In individuals with PD, such behaviors may reinforce catastrophic interpretations of bodily sensations and increase reliance on external reassurance. Consequently, high levels of CYB may complicate the therapeutic process by maintaining maladaptive metacognitive beliefs about the necessity of repeatedly checking health information online.

Within cognitive–behavioral therapy (CBT) protocols for PD, it may therefore be beneficial to address excessive online health information-seeking behaviors directly. This may include psychoeducation about the paradoxical effects of repeated online symptom searching, behavioral experiments aimed at evaluating the reliability of online medical information, and response prevention strategies targeting reassurance-seeking behaviors. Incorporating such strategies into standard CBT interventions may help reduce anxiety-related distress and facilitate the restructuring of catastrophic beliefs about bodily sensations in patients presenting with both PD and significant CYB behaviors.

Educational attainment may represent an additional factor influencing CYB-related behaviors. Individuals with different levels of education may vary in their ability to evaluate online health information critically, interpret medical content, and tolerate uncertainty, which may, in turn, influence their susceptibility to anxiety-provoking interpretations. Future studies should examine whether education level moderates the relationship between AS and CYB, particularly through its impact on e-health literacy and information-processing styles.

Future studies are needed to further clarify the mechanisms linking CYB, AS, and PD. In particular, longitudinal designs may help determine the temporal and causal relationships among these variables and clarify whether CYB contributes to the persistence or exacerbation of panic-related symptoms over time.

In addition, future research may benefit from examining the clinical effectiveness of psychological interventions specifically targeting CYB-related behaviors. For example, intervention studies could evaluate whether integrating strategies such as psychoeducation about the paradoxical effects of online health searches, behavioral experiments testing the validity of online health information, and response prevention techniques aimed at reducing reassurance-seeking behaviors may improve treatment outcomes for patients with PD who also exhibit CYB. Such approaches may be particularly relevant for CBT protocols, where addressing maladaptive online health information-seeking patterns and dysfunctional beliefs about bodily sensations could potentially reduce anxiety-related distress and improve clinical outcomes in patients with PD.

Future therapeutic research should also investigate whether patients with elevated CYB levels require specific adaptations within standard CBT protocols for PD, such as structured monitoring of online health information-seeking behaviors or targeted interventions addressing digital reassurance seeking.

## 5. Conclusions

In conclusion, this study found that patients with PD exhibited significantly higher levels of CYB and AS than healthy controls, and that these variables were positively correlated. However, PD severity appears to be primarily driven by general anxiety symptoms rather than CYB.

These findings suggest that CYB may represent a parallel maladaptive coping behavior rather than a direct determinant of panic severity. Clinically, a comprehensive assessment of patients with PD should include the evaluation of CYB and AS in addition to panic symptoms. Addressing maladaptive online health information-seeking behaviors may contribute to a more comprehensive understanding and management of panic-related processes.

## Figures and Tables

**Table 1:** Sociodemographic Characteristics of Patient and Control Groups.

Variable		Patient Mean ± SD	Control Mean ± SD	*p*-Value
Age (years)		30.21 ± 8.85	28.46 ± 7.61	0.336
		Patient *n* (%)	Control *n* (%)	*p*-value
Gender	Male	27 (38.0)	28 (40.6)	0.757
	Female	44 (62.0)	41 (59.4)	
Education level	Primary school	13 (18.3)	8 (11.6)	0.033
	High school	24 (33.8)	13 (18.8)	
	University	34 (47.9)	48 (69.6)	
Employment status	Employed	31 (43.7)	54 (78.3)	<0.001
	Unemployed	40 (56.3)	15 (21.7)	
Marital status	Single	37 (52.1)	25 (36.2)	0.09
	Married	28 (39.4)	40 (58.0)	
	Widow/Divorced	6 (8.5)	4 (5.8)	
Family structure	Nuclear	56 (78.9)	44 (63.8)	0.115
	Extended	11 (15.5)	16 (23.2)	
	Broken	4 (5.6)	9 (13.0)	
Place of residence	City	66 (93.0)	69 (100.0)	0.058
	District	5 (7.0)	0 (0.0)	
Income Level	Low	8 (11.3)	0 (0.0)	0.006
	Moderate	57 (80.3)	58 (84.1)	
	High	6 (8.5)	11 (15.9)	
Smoking	Yes	25 (35.2)	5 (7.2)	<0.001
	No	46 (64.8)	64 (92.8)	
Family history of mental illness	Yes	25 (35.2)	5 (7.2)	<0.001
	No	46 (64.8)	64 (92.8)	

**Table 2:** Distribution of Panic Attack Symptoms in the Patient Group.

Symptom	Status	*n* (%)
Palpitations	Yes	69 (97.2)
	No	2 (2.8)
Sweating	Yes	56 (78.9)
	No	15 (21.1)
Shaking/trembling	Yes	51 (71.8)
	No	20 (28.2)
Shortness of breath/feeling of suffocation	Yes	59 (83.1)
	No	12 (16.9)
Sensation of breathlessness	Yes	55 (77.5)
	No	16 (22.5)
Chest pain	Yes	50 (70.4)
	No	21 (29.6)
Nausea/stomach ache	Yes	38 (53.5)
	No	33 (46.5)
Dizziness/lightheadedness	Yes	44 (62.0)
	No	27 (38.0)
Hot flashes/chills	Yes	49 (69.0)
	No	22 (31.0)
Numbness/tingling	Yes	44 (62.0)
	No	27 (38.0)
Derealization/depersonalization	Yes	33 (46.5)
	No	38 (53.5)
Fear of losing control/going crazy	Yes	47 (66.2)
	No	24 (33.8)
Fear of death	Yes	52 (73.2)
	No	19 (26.8)

**Table 3:** Adjusted comparisons of patients and control groups (ANCOVA results).

Variable	Patient (Adjusted Mean ± SD)	Control (Adjusted Mean ± SD)	F	*p*-Value	Effect Size (η^2^)	Covariate Effects
ASI-3 Physical	13.56 ± 0.69	4.49 ± 0.70	71.23	<0.001	0.349	Not significant (all *p* > 0.05)
ASI-3 Cognitive	13.03 ± 0.82	6.36 ± 0.37	19.57	<0.001	0.128	Employment status *p* = 0.020; Smoking *p* = 0.041
ASI-3 Social	7.88 ± 0.63	3.64 ± 0.65	18.07	<0.001	0.12	Not significant (all *p* > 0.05)
ASI-3 Total	33.47 ± 1.88	14.49 ± 1.92	41.57	<0.001	0.238	Not significant (all *p* > 0.05)
CSS-Compulsion	14.85 ± 0.75	10.87 ± 0.76	11.61	<0.001	0.08	Smoking *p* = 0.002; others *p* > 0.05
CSS-Distress	21.18 ± 0.92	14.63 ± 0.93	21.05	0.001	0.137	Not significant (all *p* > 0.05)
CSS-Excessiveness	22.20 ± 0.95	17.64 ± 0.96	9.48	0.003	0.067	Not significant (all *p* > 0.05)
CSS–Reassurance	12.13 ± 0.60	11.83 ± 0.61	0.09	0.753	0.001	Smoking *p* = 0.003; others *p* > 0.05
CSS–Mistrust	7.75 ± 0.50	8.31 ± 0.51	0.55	0.46	0.004	Family history of mental illness *p* = 0.007; others *p* > 0.05
CSS–Total	78.11 ± 2.62	63.29 ± 2.67	13.01	<0.001	0.089	Smoking *p* = 0.033; others *p* > 0.05
BAI–Total	32.63 ± 1.42	7.22 ± 1.44	131.82	<0.001	0.52	Not significant (all *p* > 0.05)

ASI-3 = Anxiety Sensitivity Index-3; CSS = Cyberchondria Severity Scale; BAI = Beck Anxiety Inventory; PDSS = Panic Disorder Severity Scale. ASI-3 Physical, Cognitive, and Social represent the respective subscales of the Anxiety Sensitivity Index-3. CSS-Compulsion, Distress, Excessiveness, Reassurance, and Mistrust represent the corresponding subscales of the Cyberchondria Severity Scale. η^2^ = partial eta squared (effect size). Values of 0.01, 0.06, and 0.14 indicate small, medium, and large effect sizes, respectively.

**Table 4:** Correlations Between Anxiety Sensitivity, Cyberchondria, and Anxiety and Panic Disorder Severity Scale Scores in the Patient Group.

Variable	1	2	3	4	5	6	7	8	9	10	11	12
1. ASI-3 Physical	—											
2. ASI-3 Cognitive	0.666 **	—										
3. ASI-3 Social	0.561 **	0.758 **	—									
4. ASI-3 Total	0.846 **	0.927 **	0.866 **	—								
5. CSS-Compulsion	0.21	0.329 **	0.368 **	0.342 **	—							
6. CSS-Distress	0.415 **	0.365 **	0.297 *	0.410 **	0.622 **	—						
7. CSS-Excessiveness	0.285 *	0.217	0.251 *	0.283 *	0.516 **	0.588 **	—					
8. CSS-Reassurance	0.164	0.115	0.177	0.169	0.538 **	0.497 **	0.417 **	—				
9. CSS-Mistrust	−0.053	−0.002	−0.050	−0.038	0.163	0.003	−0.138	−0.028	—			
10. CSS-Total	0.336 **	0.333 **	0.337 **	0.380 **	0.853 **	0.859 **	0.759 **	0.696 **	0.158	—		
11. BAI-Total	0.667 **	0.655 **	0.591 **	0.726 **	0.119	0.17	0.179	0.055	−0.15	0.15	—	
12. PDSS-Total	0.424 **	0.425 **	0.369 **	0.463 **	0.161	0.094	0.059	−0.002	−0.100	0.09	0.571 **	—

ASI-3 = Anxiety Sensitivity Index-3; CSS = Cyberchondria Severity Scale; BAI = Beck Anxiety Inventory; PDSS = Panic Disorder Severity Scale. ASI-3 Physical, ASI-3 Cognitive, and ASI-3 Social represent the physical, cognitive, and social subscales of the Anxiety Sensitivity Index-3, respectively. CSS-Compulsion, CSS-Distress, CSS-Excessiveness, CSS-Reassurance, and CSS-Mistrust represent the corresponding subscales of the Cyberchondria Severity Scale. * *p* < 0.05; ** *p* < 0.01.

**Table 5:** Hierarchical Regression Analysis of Variables Predicting Panic Disorder Severity Score (PDSS-Total) in a Patient Group.

**Model 1**										
**Variable**	**β**	**SE**	**t**	***p***	**VIF**	**R^2^**	**Adjusted R^2^**	**ΔR^2^**	**F**	***p*** **-Value**
Age	−0.245	0.115	−1.975	0.052	1.124	0.098	0.044	0.098	1.8	0.139
Employment status	0.037	2.01	0.303	0.763	1.091					
Smoking	0.207	2.011	1.756	0.084	1.013					
Family history of mental illness	0.041	2.042	0.343	0.733	1.045					
**Model 2**										
**Variable**	**β**	**SE**	**t**	***p***	**VIF**	**R^2^**	**Adjusted R^2^**	**ΔR^2^**	**F**	***p*** **-Value**
Age	−0.25	0.116	−1.999	0.05	1.131	0.102	0.033	0.004	1.478	0.209
Employment status	0.036	2.021	0.293	0.77	1.091					
Smoking	0.192	2.078	1.579	0.119	1.07					
Family history of mental illness	0.035	2.063	0.29	0.773	1.055					
CSS-Total	0.063	0.044	0.518	0.606	1.068					
**Model 3**										
**Variable**	**β**	**SE**	**t**	***p***	**VIF**	**R^2^**	**Adjusted R^2^**	**ΔR^2^**	**F**	***p*** **-Value**
Age	−0.215	0.105	−1.895	0.063	1.138	0.279	0.212	0.177	4.136	0.001 **
Employment status	0.122	1.859	1.082	0.283	1.133					
Smoking	0.104	1.914	0.927	0.357	1.114					
Family history of mental illness	0.031	1.863	0.281	0.78	1.055					
CSS-Total	−0.104	0.043	−0.886	0.379	1.226					
ASI−3 Total	0.477	0.055	3.969	<0.001	1.286					
**Model 4**										
**Variable**	**β**	**SE**	**t**	***p***	**VIF**	**R^2^**	**Adjusted R^2^**	**ΔR^2^**	**F**	***p*** **-Value**
Age	−0.179	0.099	−1.679	0.098	1.151	0.379	0.31	0.1	5.496	<0.001 **
Employment status	0.175	1.761	1.635	0.107	1.161					
Smoking	0.065	1.803	0.615	0.54	1.129					
Family history of mental illness	−0.002	1.751	−0.019	0.985	1.065					
CSS-Total	−0.034	0.041	−0.307	0.76	1.274					
ASI-3 Total	0.123	0.073	0.776	0.441	2.546					
BAI-Total	0.486	0.087	3.181	0.002	2.365					

CSS-Total = total score of the Cyberchondria Severity Scale; ASI-3 Total = total score of the Anxiety Sensitivity Index-3; BAI-Total = total score of the Beck Anxiety Inventory; PDSS-Total = total score of the Panic Disorder Severity Scale. β = standardized regression coefficient; SE = standard error; VIF = variance inflation factor. R^2^ = coefficient of determination; ΔR^2^ = change in R^2^. All regression models were adjusted for age, employment status, smoking status, and family history of mental illness. No multicollinearity was detected (VIF < 5). ** *p* < 0.01.

**Table 6:** Comparison of cyberchondria scores according to agoraphobia status in the patient group.

Variable	Without Agoraphobia (*n* = 48), Mean ± SD	With Agoraphobia (*n* = 23), Mean ± SD	t	*p*-Value	Mean Difference (95% CI)
CSS–Compulsion	15.65 ± 7.80	14.70 ± 6.62	0.50	0.616	0.95 (−2.81, 4.71)
CSS–Distress	21.44 ± 8.88	23.26 ± 7.34	−0.85	0.396	−1.82 (−6.08, 2.43)
CSS–Excessiveness	22.58 ± 7.19	23.26 ± 6.49	−0.38	0.703	−0.68 (−4.21, 2.85)
CSS–Reassurance	13.23 ± 4.94	12.48 ± 4.72	0.61	0.545	0.75 (−1.71, 3.22)
CSS–Mistrust	7.79 ± 3.29	7.04 ± 3.80	0.85	0.396	0.75 (−1.00, 2.50)
CSS–Total	80.69 ± 24.19	80.74 ± 19.77	−0.01	0.993	−0.05 (−11.62, 11.52)

CSS = Cyberchondria Severity Scale. Values are presented as mean ± standard deviation. Group comparisons were performed using independent samples *t*-tests.

## Data Availability

The data supporting the findings of this study contain sensitive patient information and are therefore not publicly available. Anonymized data may be made available from the corresponding author upon reasonable request.

## Abbreviations

The following abbreviations are used in this manuscript:

PD	Panic Disorder
CYB	Cyberchondria
AS	Anxiety Sensitivity
CSS	Cyberchondria Severity Scale
ASI-3	Anxiety Sensitivity Index-3
BAI	Beck Anxiety Inventory
PDSS	Panic Disorder Severity Scale
DSM-5	Diagnostic and Statistical Manual of Mental Disorders, Fifth Edition
SCID-5-CV	Structured Clinical Interview for DSM-5, Clinician Version
CBT	Cognitive Behavioral Therapy
